# MCEE Promotes Intramuscular Fat Deposition in Pigs Through Regulating Mitochondrial Function

**DOI:** 10.3390/ani15192797

**Published:** 2025-09-25

**Authors:** Yasai Li, Xinyue Chen, Dake Chen, Junjing Wu, Tong Chen, Mu Qiao, Xianwen Peng, Shuqi Mei, Yue Feng

**Affiliations:** 1 Hubei Key Laboratory of Animal Embryo and Molecular Breeding, Institute of Animal Husbandry and Veterinary, Hubei Academy of Agricultural Sciences, Wuhan 430064, China; lyslys0811@163.com (Y.L.);; 2 College of Animal Science, Yangtze University, Jingzhou 434025, China; xinyue_ccc@163.com; 3 Hubei Hongshan Laboratory, Wuhan 430070, China

**Keywords:** pig, *MCEE*, intramuscular fat, meat quality

## Abstract

IMF is a key determinant of meat quality, influencing tenderness, juiciness and flavor, yet the mechanisms underlying its formation remain poorly understood. Previous studies performed whole-genome resequencing and GWAS on pigs with divergent IMF content, identifying *MCEE* as a candidate gene associated with IMF deposition. Subsequently, gain- and loss-of-function approaches were employed to investigate the role of *MCEE* in porcine intramuscular preadipocytes. Here, we isolated primary preadipocytes and subjected them to adipogenic induction. The overexpression of *MCEE* enhanced the proliferation and adipogenic differentiation of porcine intramuscular preadipocytes, whereas its knockdown exerted the opposite effect. Transcriptomic analysis revealed that DEGs were primarily enriched in pathways related to oxidative phosphorylation, mitochondrial dysfunction-associated disorders and others. Subcellular localization prediction indicated mitochondrial targeting of *MCEE*, and its expression level influenced mitochondrial function, including reactive oxygen species levels, mitochondrial membrane potential and permeability transition pore opening. Collectively, *MCEE* regulates IMF deposition by modulating mitochondrial function, and these findings provide a potential molecular target for improving meat quality.

## Simple Summary

Intramuscular fat (IMF) content is one of the key factors influencing pork quality. Previous whole-genome resequencing and genome-wide association study (GWAS) analyses identified methylmalonyl-CoA epimerase enzyme (*MCEE*) as a candidate gene associated with porcine IMF. Experimental validation revealed that *MCEE* overexpression in porcine preadipocytes promotes cell proliferation and adipogenic differentiation, whereas its suppression exerts the opposite effect. Transcriptome profiling of *MCEE*-overexpressing and knockdown cells demonstrated that differentially expressed genes (DEGs) were primarily enriched in pathways related to oxidative phosphorylation and mitochondrial dysfunction-related disorders. Further experiments indicated that *MCEE* overexpression enhances mitochondrial function, while its inhibition impairs it. These findings suggest that *MCEE* regulates IMF deposition by modulating mitochondrial function, providing a potential target for improving livestock meat quality, especially pork meat quality.

## 1. Introduction

Pork is the most consumed meat category in China, and improving meat quality is crucial to meet the growing demand for high-quality protein driven by population growth and economic development [1]. Intramuscular fat (IMF), a key determinant of pork quality, directly influences tenderness, juiciness, and flavor characteristics through its content and distribution [2]. IMF exists as lipid droplets within muscle fibers and the surrounding perimysial connective tissue, primarily composed of triglycerides (TGs), along with minor amounts of cholesterol and phospholipids [3]. By loosening connective tissue structure, IMF reduces muscle shear force, significantly enhancing tenderness while also affecting nutritional and economic value [4]. Studies demonstrate a strong positive correlation between IMF content and tenderness, with higher IMF levels yielding improved meat tenderness after cooking [5]. However, selective breeding for leaner pig breeds (such as Landrace, Large White and Duroc) has led to declining IMF content, resulting in poorer meat quality and reduced consumer acceptance [6]. Furthermore, free fatty acids released from TG hydrolysis participate in Maillard reactions to generate pyrazines and furanones, while oxidative degradation of phospholipids produces aldehydes and ketones that form the backbone of meat aroma [7]. The ratio of saturated fatty acids (SFAs), monounsaturated fatty acids (MUFAs), and polyunsaturated fatty acids (PUFAs) in IMF also significantly influences the nutritional health value of meat [8]. Precise regulation of IMF formation can optimize livestock production efficiency, enhance market competitiveness, and generate substantial economic benefits for farmers, producers, and consumers.

The process of adipogenesis and lipid accumulation is intrinsically linked to mitochondrial function. Mitochondria are dynamic organelles that undergo constant biogenesis, fission, and fusion to meet cellular energy demands and regulate metabolic pathways. Recent studies in livestock species have highlighted the critical role of mitochondrial activity in adipocyte differentiation and lipid metabolism. In bovine adipocytes, m the interaction between lipid regulatory elements and cell surface proteins like CD44 has been shown to modulate mitochondrial bioenergetics, thereby influencing adipogenic differentiation and lipid droplet composition [9]. Similarly, research on Sirtuin 5 (*SIRT5*) in cattle revealed its profound impact on mitochondrial function during preadipocyte differentiation, where it helps maintain mitochondrial integrity and regulates the levels of various lipid metabolites, ultimately affecting triglyceride accumulation and fatty acid profiles [10]. In pigs, the leucine metabolite β-hydroxy-β-methylbutyrate (*HMB*) has been shown to modulate lipid metabolism in adipose tissue, partly through mechanisms involving the AMPK pathway, a central regulator of cellular energy status and mitochondrial biogenesis [11]. These findings collectively underscore that mitochondrial regulatory mechanisms are fundamental to understanding fat deposition in livestock species.

The methylmalonyl-CoA epimerase (*MCEE*) gene belongs to the vicinal oxygen chelate (VOC) superfamily and encodes a monomeric protein characterized by an α/β-fold structure composed of two glyoxalase domains [12]. As a key enzyme, MCEE plays a central role in the catabolism of odd-chain fatty acids (OCFAs) and certain amino acids such as valine, isoleucine, and methionine [12,13]. OCFAs, which contain an odd number of carbon atoms, yield propionyl-CoA as the end product of β-oxidation. MCEE connects propionyl-CoA metabolism to the tricarboxylic acid (TCA) cycle by catalyzing the stereoisomeric conversion of methylmalonyl-CoA, thereby playing a critical regulatory role in energy metabolism homeostasis [14]. Studies have shown that the overexpression of MCEE significantly enhances the conversion efficiency of propionate to pyruvate and promotes hepatic gluconeogenesis in cattle, a process finely regulated by the MTORC1–PGC1α signaling pathway [15]. Notably, L-methylmalonyl-CoA, the sole substrate of methylmalonyl-CoA mutase (MUT), is further converted to succinyl-CoA, which enters the TCA cycle for ATP production [14]. Although the content of OCFAs in muscle tissue is generally lower than in adipose tissue [16], and OCFAs are stored in small amounts in adipose tissue, their overall metabolism tends to be catabolic [17]. In summary, *MCEE* may influence intramuscular fat deposition by regulating the integration of propionyl-CoA metabolism with the TCA cycle, thereby modulating energy production and lipid metabolism dynamics.

This study demonstrates that *MCEE* plays a pivotal role in regulating IMF deposition by controlling the proliferation and adipogenic differentiation of porcine intramuscular preadipocytes, with its overexpression promoting adipogenesis and knockdown suppressing it. Transcriptomic and functional analyses reveal that *MCEE* modulates mitochondrial function, including reactive oxygen species levels, mitochondrial membrane potential, and permeability transition pore opening. These findings indicate that *MCEE* regulates porcine IMF by influencing mitochondrial activity, providing a promising molecular target for improving pork quality.

## 2. Materials and Methods

### 2.1. Isolation, Culture and Differentiation of Preadipocytes

Intramuscular preadipocytes were isolated following established protocols [18]. Three-day-old piglets were obtained from a commercial pig farm in Hubei Province. Under sterile conditions, the middle section of the longissimus dorsi muscle was immediately excised and digested with 2 mg/mL type II collagenase solution (Invitrogen, Waltham, MA, USA) (2× tissue volume) at 37 °C with constant agitation for 80 min. The digested tissue was sequentially filtered through 100 μm, 70 μm, and 40 μm cell strainers. The filtrate was centrifuged at 1000 rpm for 5 min to collect cell pellets, which were then resuspended in 5 volumes of 1× erythrocyte lysis buffer, gently mixed, and incubated at 4 °C for 2 min. After centrifugation at 1000 rpm for 5 min, the supernatant was carefully removed. The cell pellet was washed twice with PBS and resuspended in DMEM-F12 medium (Gibco, Grand Island, NY, USA) supplemented with 10% fetal bovine serum (Gibco), followed by culture at 37 °C with 5% CO_2_.

### 2.2. Immunofluorescence Staining

Immunofluorescence staining was performed when porcine intramuscular preadipocytes reached 80–90% confluence. The procedure was conducted as follows: Culture medium was removed and cells were washed twice with PBS (5 min per wash). Cells were fixed with 4% paraformaldehyde (Beyotime, Shanghai, China) at room temperature for 15 min, followed by three PBS washes (5 min each). Permeabilization was performed using immunostaining Permeabilization Buffer with 0.2% Triton X-100 (P0096, Beyotime, Shanghai, China) for 30 min, with subsequent three PBS washes (5 min each). After blocking with immunostaining blocking buffer for 30 min, 200 μL of primary antibody was added to each well and incubated overnight at 4 °C. Cells were washed three times with PBS (5 min each). Fluorescent secondary antibody was added and incubated at 37 °C in the dark for 2 h, followed by three PBS washes (5 min each). Nuclei were stained with DAPI (Beyotime, Shanghai, China) in the dark for 5 min, with three subsequent PBS washes (5 min each). Finally, antifade mounting medium (Beyotime, Shanghai, China) was added in the dark, and samples were observed and imaged using an inverted fluorescence microscope (Nikon Instruments, Melville, NY, USA).

### 2.3. Plasmid Construction, Interference Fragment Synthesis and Cell Transfection

The coding sequence (CDS) region of *MCEE* was amplified from porcine cDNA and cloned into the pcDNA3.1 overexpression vector using *Hin*d III (ABclonal, Wuhan, China) and *Xba* I (ABclonal, Wuhan, China) restriction sites through homologous recombination (ABclonal, Wuhan, China). siRNA targeting *MCEE* (*MCEE* siRNA) and negative control (siRNA-NC) were designed and synthesized by GenePharma (Suzhou, China). Prior to transfection, cells were cultured to 70–80% confluence. The overexpression vector pcDNA3.1-*MCEE* and siRNA interference fragments were transfected into cells using Lipofectamine RNAiMAX (Invitrogen, Waltham, MA, USA), while plasmids were transfected using Lipofectamine 3000 (Invitrogen, Waltham, MA, USA).

### 2.4. BODIPY Staining and Oil Red O Staining

Porcine intramuscular preadipocytes were transfected with either the pcDNA3.1-*MCEE* overexpression vector or siRNA interference fragments and were subjected to the following experimental procedures on day 8 of adipogenic differentiation induction. Cells were fixed with 1 mL of 4% paraformaldehyde at room temperature for 30 min. For BODIPY staining, cells were incubated with 0.5 nM BODIPY™ 493/503 (Invitrogen, Waltham, MA, USA) for 15 min; for Oil Red O staining, cells were stained with Oil Red O working solution (Sigma-Aldrich, St. Louis, MO, USA) for 30 min. Images were acquired using fluorescence microscopy, and mean fluorescence intensity was quantified using ImageJ software version 1.53.

### 2.5. RNA Extraction and Real-Time Quantitative PCR

Total cellular RNA was extracted using TRIzol reagent (Invitrogen, Waltham, MA, USA). Complementary DNA (cDNA) was synthesized using a cDNA synthesis kit (Thermo Fisher Scientific, Waltham, MA, USA). Quantitative real-time PCR (qRT-PCR) was performed using the QuantStudio 6 Flex Real-Time PCR System (Applied Biosystems, Foster City, CA, USA) with iTaq Universal SYBR Green Supermix (Bio-Rad, Richmond, CA, USA). RT-qPCR primers are listed in Table 1. Gene expression levels were normalized to β-actin expression using the 2^−ΔΔCt^ method.

### 2.6. Western Blotting

Total cellular proteins were extracted using RIPA lysis buffer (Beyotime, Shanghai, China). Protein concentrations were quantified using a BCA protein assay kit (P0012S, Beyotime, Shanghai, China) according to the manufacturer’s protocol. Proteins were then separated by SDS-PAGE and transferred onto polyvinylidene difluoride (PVDF) membranes (Millipore, Billerica, MA, USA) using a Mini Trans-Blot system (Bio-Rad, Richmond, CA, USA). Immunoblotting was performed using the following primary antibodies: anti-MCEE (1:500; ABclonal, Wuhan, China), anti-β-actin (1:100,000; ABclonal, Wuhan, China), and anti-FABP4 (1:1000; ABclonal, Wuhan, China). Protein expression was detected using an Image Quant LAS4000 mini system (GE Healthcare Life Sciences, Piscataway, NJ, USA), and band intensities were quantified using ImageJ software.

### 2.7. CCK-8 Assay

Porcine intramuscular preadipocytes were seeded in 96-well plates and transfected with either the pcDNA3.1-*MCEE* overexpression vector or siRNA interference fragments. 24 h post-transfection, cell differentiation was induced. Cell proliferation was assessed at 0, 12, 24, 36, and 48 h after differentiation induction using the CCK-8 assay. The experimental procedure was as follows: CCK-8 reagent (Beyotime, Shanghai, China) (10 μL) was added 2 h prior to measurement, and absorbance was measured at 450 nm using a microplate reader (PerkinElmer, Waltham, MA, USA). Relative proliferation rates were calculated to assess *MCEE*’s effect on cell proliferation.

### 2.8. Apoptosis and Cell Cycle Analysis by Flow Cytometry

Porcine intramuscular preadipocytes were transfected with either pcDNA3.1-*MCEE* overexpression vector or *MCEE* siRNA interference fragments. On day 2 of adipogenic differentiation induction, the cells were digested, collected and processed for flow cytometric analysis to determine cell cycle distribution and apoptosis rates.

### 2.9. Subcellular Localization Prediction

The amino acid sequence of *MCEE* was retrieved from the NCBI (https://www.ncbi.nlm.nih.gov/ accessed on 15 July 2025) database, and its subcellular localization was predicted using DeepLoc-2.1 (https://services.healthtech.dtu.dk/services/DeepLoc-2.0/ accessed on 6 March 2025) and Cell-PLoc 2.0 (http://www.csbio.sjtu.edu.cn/bioinf/Cell-PLoc-2/ accessed on 10 April 2025).

### 2.10. Reactive Oxygen Species (ROS) Detection

Porcine intramuscular preadipocytes were transfected with either pcDNA3.1-*MCEE* overexpression vector or *MCEE* siRNA interference fragments. On day 2 of adipogenic differentiation induction, reactive oxygen species (ROS) were detected as follows: Cells were loaded with the DCFH-DA fluorescent probe (Sigma-Aldrich, St. Louis, MO, USA) (10 μM final concentration, diluted 1:1000 in PBS). After removing culture medium, cells were incubated with DCFH-DA solution for 20 min at 37 °C, then washed three times with serum-free medium. Fluorescence was measured at 485 nm excitation/525 nm emission using a microplate reader (PerkinElmer, Waltham, MA, USA). Fluorescence images were analyzed with ImageJ software, with fluorescence intensity being proportional to intracellular ROS levels.

### 2.11. Mitochondrial Membrane Potential (ΔΨm) Assay

Porcine intramuscular preadipocytes were transfected with either pcDNA3.1-*MCEE* overexpression vector or *MCEE* siRNA interference fragments, followed by 2 days of adipogenic differentiation induction. On day 2 of differentiation, mitochondrial membrane potential was assessed using the JC-1 assay kit (Beyotime, Shanghai, China) according to the manufacturer’s protocol. The degree of mitochondrial depolarization was quantified by measuring the relative ratio of red (JC-1 aggregates) to green (JC-1 monomer) fluorescence. Briefly, cells were incubated with JC-1 working solution at 37 °C for 20 min, washed three times with PBS, and imaged using fluorescence microscopy. Fluorescence intensities were analyzed with ImageJ software.

### 2.12. Mitochondrial Permeability Transition Pore (mPTP) Assay

Porcine intramuscular preadipocytes were transfected with either pcDNA3.1-*MCEE* overexpression vector or *MCEE* siRNA interference fragments, followed by 2 days of adipogenic differentiation induction. On day 2 of differentiation, mitochondrial permeability transition pore (mPTP) opening was assessed using the MPTP Detection Kit (Beyotime, Shanghai, China) according to the manufacturer’s protocol. The degree of mPTP opening was quantified by measuring calcein green fluorescence intensity in mitochondria. Briefly, cells were incubated with calcein AM staining solution at 37 °C for 30 min, followed by replacement with fresh pre-warmed culture medium and additional 30 min incubation in the dark. After three PBS washes, green fluorescence signals were captured by fluorescence microscopy and analyzed using ImageJ software.

### 2.13. RNA-Seq

Porcine intramuscular preadipocytes were transfected with pcDNA3.1-*MCEE* overexpression vector or *MCEE* siRNA interference fragments and induced for adipogenic differentiation for 2 days. On day 2 of differentiation, sequencing was performed using the PE150 strategy on the Illumina platform (Novogene, Beijing, China). Raw sequencing reads were quality-assessed using FastQC (v0.11.9) and processed with Trimmomatic (v0.39) to generate high-quality clean reads. These clean reads were then aligned to the Sus scrofa 11.1 reference genome using HISAT2 (v2.2.1). Differential gene expression analysis was performed using DESeq2 (v1.26.0) with thresholds set at *p*-value < 0.05 and |log_2_FoldChange| > 2 to identify significantly differentially expressed genes. Three biological replicates were included in this analysis.

### 2.14. Gene Annotation and Functional Analysis

In this study, the clusterProfiler software (v3.18.1) was employed to perform Gene Ontology (GO) functional enrichment analysis and KEGG pathway enrichment analysis on the differentially expressed gene set obtained from significant differential expression analysis. The screening threshold was set at *p* < 0.05.

### 2.15. Statistical Analysis

All results are presented as mean ± standard deviation. Comparisons between two groups were performed using two-tailed *t*-tests with three biological replicates per treatment. Significant differences were determined by independent-sample *t*-tests, with *p*-values < 0.05 were considered statistically significant (*), *p*-values < 0.01 were considered highly statistically significant (**) and *p*-values < 0.001 were considered highly statistically significant (***).

## 3. Results

### 3.1. The Expression Pattern Analysis of MCEE

**Figure 1:**
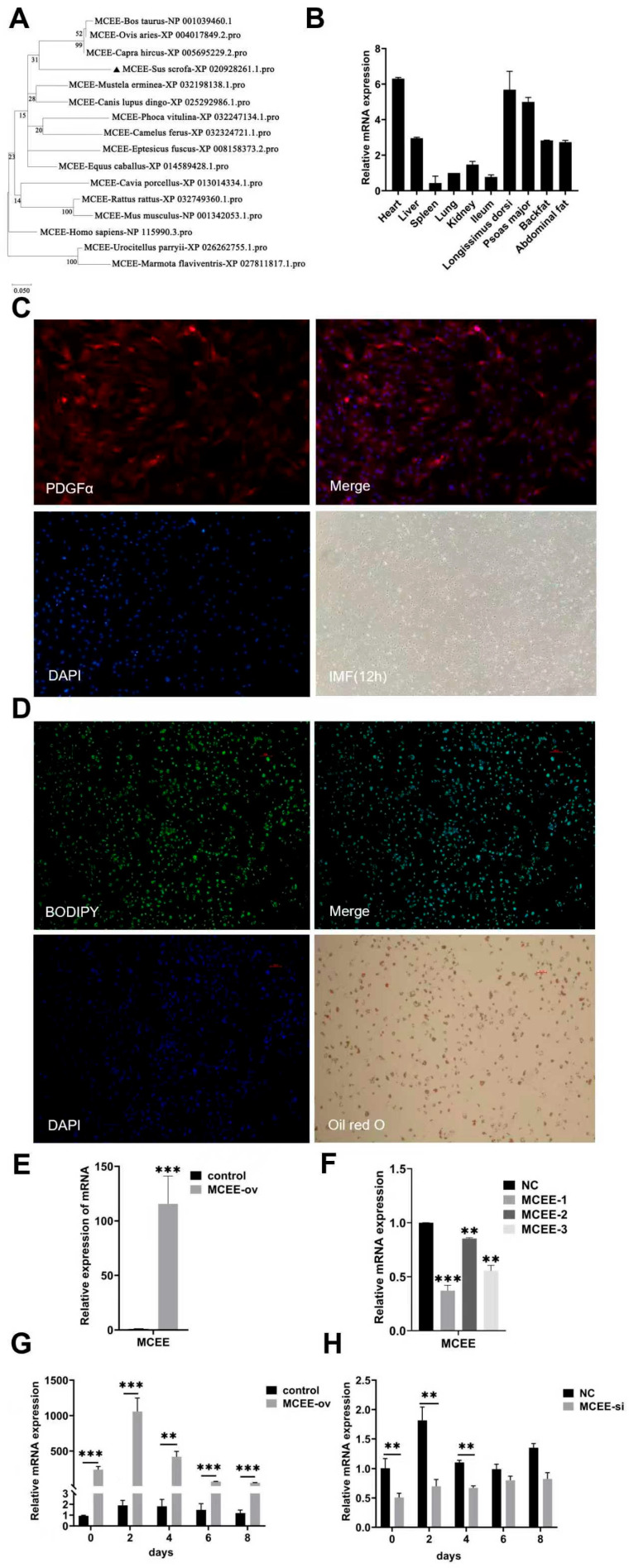
Analysis of *MCEE* expression patterns. (**A**) Phylogenetic tree of *MCEE*. The branch marked with a black triangle represents the pig breed (Sus scrofa). (**B**) Tissue expression profile of *MCEE*. (**C**) PDGFα immunofluorescence staining in isolated porcine intramuscular preadipocytes following differentiation induction. (**D**) Assessment of lipid droplet formation in differentiated preadipocytes using BODIPY and Oil Red O staining. (**E**) Quantitative detection of *MCEE* mRNA expression levels upon overexpression. (**F**) Quantitative detection of *MCEE* mRNA expression levels upon knockdown (**G**) Temporal analysis of *MCEE* mRNA expression during adipogenic differentiation following *MCEE* overexpression. (**H**) Temporal analysis of *MCEE* mRNA expression during adipogenic differentiation following MCEE knockdown. ** *p* < 0.01, *** *p* < 0.001.

To investigate the evolutionary relationships of *MCEE* among species, we constructed a phylogenetic tree revealing that porcine *MCEE* clusters with bovine, ovine and caprine orthologs, indicating high sequence similarity and close evolutionary relationships. Distinct clustering was also observed between primates (human) and rodents (mouse) (Figure 1A). Tissue expression profiling across ten porcine tissues demonstrated ubiquitous *MCEE* expression, with highest levels in cardiac tissue, followed by longissimus dorsi and lumbar muscles, while spleen, lung and ileum showed lower expression (Figure 1B). We isolated intramuscular preadipocytes from 3-day-old piglets. Immunofluorescence staining confirmed high purity of the isolated cell population, with PDGFα-positive cells representing >90% of the total population (Figure 1C). The adipogenic differentiation capacity was verified by BODIPY and Oil Red O staining (Figure 1D). Quantitative analysis revealed that *MCEE* expression was significantly upregulated in the pcDNA3.1-*MCEE* overexpression group compared with the control group (Figure 1E), whereas *MCEE* siRNA knockdown groups exhibited markedly reduced *MCEE* expression, with siRNA-1 demonstrating the highest knockdown efficiency (Figure 1F). During adipogenic differentiation (days 0–8), both overexpression and knockdown efficiencies peaked at day 2 post-induction (Figure 1G,H).

### 3.2. Overexpression of MCEE Promotes the Proliferation and Adipogenic Differentiation of Porcine Intramuscular Adipocytes

**Figure 2:**
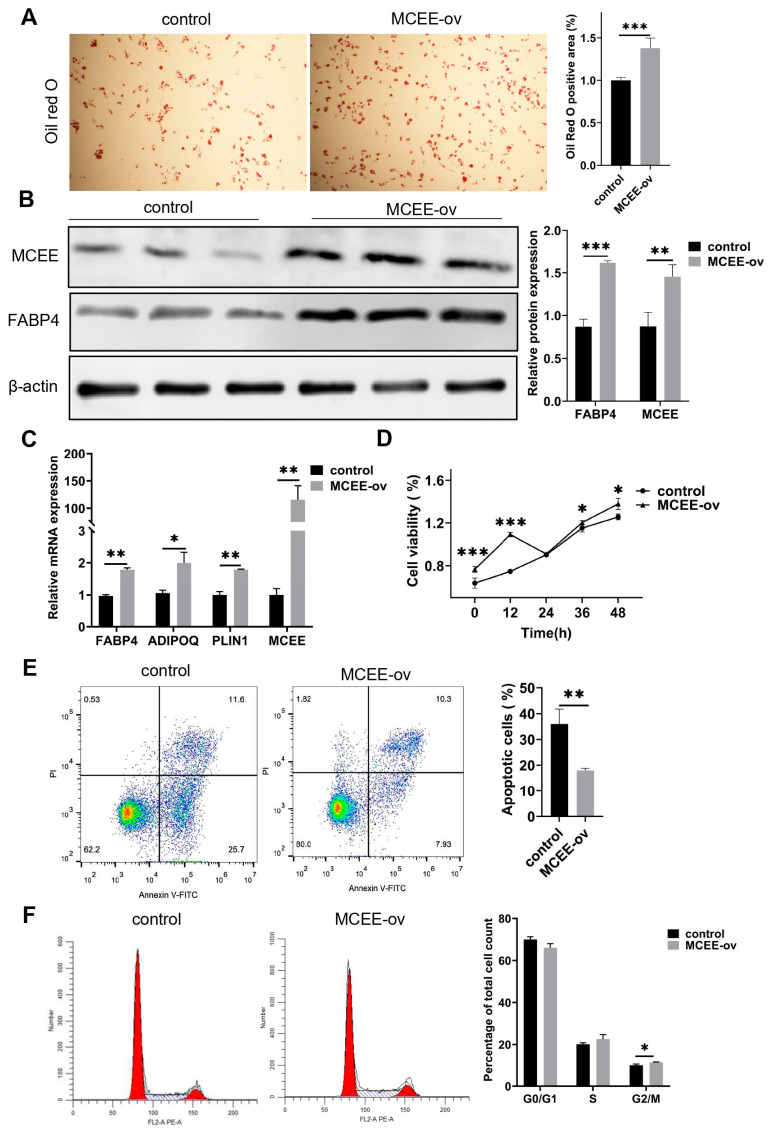
Effects of *MCEE* overexpression on proliferation and differentiation of intramuscular adipocytes. (**A**) Oil Red O staining of *MCEE*-overexpressing cells on day 8 of differentiation induction. Left panel: representative Oil Red O staining. Right panel: quantification of Oil Red O staining. (**B**) Western blot analysis of MCEE and FABP4 protein expression levels on day 2 of differentiation induction. Left panel: representative Western blot. Right panel: quantification of Western blot. (**C**) Quantitative detection of adipogenic marker genes (*FABP4*, *ADIPOQ*, *PLIN1*) on day 2 of differentiation induction. (**D**) CCK-8 assay measuring viability of porcine intramuscular preadipocytes. (**E**) Flow cytometry analysis of apoptosis rate in porcine intramuscular preadipocytes. Lower panel: quantification of apoptosis. (**F**) Flow cytometry analysis of cell cycle distribution in porcine intramuscular preadipocytes. Lower panel: quantification of cell cycle phases. * *p* < 0.05, ** *p* < 0.01, *** *p* < 0.001.

To investigate the effect of *MCEE* overexpression on the adipogenic differentiation of intramuscular preadipocytes, we transfected cells with the overexpression vector pcDNA3.1-*MCEE*. After 8 days of adipogenic induction, Oil Red O staining revealed a significant increase in lipid droplet accumulation in the overexpression group compared to control group (Figure 2A). Quantitative analysis at day 2 of adipogenic induction demonstrated elevated FABP4 protein expression in the overexpression group (Figure 2B and Supplementary Material), accompanied by significantly increased mRNA levels of *FABP4*, *ADIPOQ*, and *PLIN1* (Figure 2C). CCK-8 assays conducted at 0, 12, 24, 36, and 48 h post-induction demonstrated significantly enhanced cell viability in the overexpression group at all timepoints except 24 h (Figure 2D). Flow cytometry analysis revealed both reduced apoptosis rates (Figure 2E) and increased G2/M phase cell populations (Figure 2F) in *MCEE*-overexpressing cells. These findings demonstrate that overexpression *MCEE* promotes proliferation and adipogenic differentiation of intramuscular preadipocytes.

### 3.3. Inhibition of MCEE Suppresses the Proliferation and Adipogenic Differentiation of Porcine Intramuscular Adipocytes

**Figure 3:**
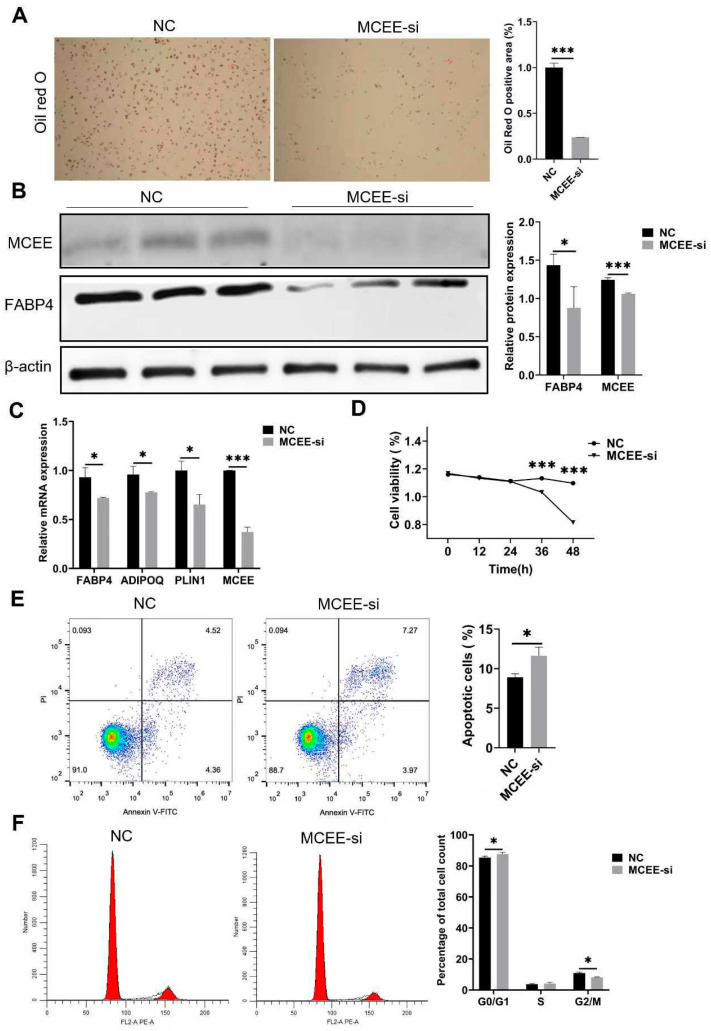
Effects of *MCEE* inhibition on proliferation and differentiation of intramuscular adipocytes. (**A**) Oil Red O staining of *MCEE*-suppressed cells on day 8 of differentiation induction. Left panel: representative Oil Red O staining. Right panel: quantification of Oil Red O staining. (**B**) Western blot analysis of MCEE and FABP4 protein expression levels on day 2 of differentiation induction. Left panel: representative Western blot. Right panel: quantification of Western blot. (**C**) Quantitative detection of adipogenic marker genes (*FABP4*, *ADIPOQ*, *PLIN1*) on day 2 of differentiation induction. (**D**) CCK-8 assay measuring viability of porcine intramuscular preadipocytes. (**E**) Flow cytometry analysis of apoptosis rate in porcine intramuscular preadipocytes. Lower panel: quantification of apoptosis. (**F**) Flow cytometry analysis of cell cycle distribution in porcine intramuscular preadipocytes. Lower panel: quantification of cell cycle phases. * *p* < 0.05, *** *p* < 0.001.

To investigate the effects of *MCEE* knockdown on adipogenic differentiation of intramuscular preadipocytes, we transfected *MCEE* siRNA into intramuscular adipocytes. Following 8 days of adipogenic induction, Oil Red O staining revealed that *MCEE* suppression significantly reduced lipid droplet accumulation compared with the control group (Figure 3A). Quantitative analysis conducted on day 2 of adipogenic induction demonstrated a marked decrease in FABP4 protein expression levels in the knockdown group (Figure 3B and Supplementary Material), along with significantly reduced mRNA expression of *FABP4*, *ADIPOQ*, and *PLIN1* (Figure 3C). CCK-8 assays performed at 0, 12, 24, 36, and 48 h post-induction indicated that cell viability was significantly lower in the *MCEE* knockdown group at 36 and 48 h (Figure 3D). Flow cytometry analysis showed that the apoptosis rate was significantly increased in the *MCEE* knockdown group compared with control group (Figure 3E), while the proportion of G2/M-phase cells was notably reduced (Figure 3F). These results suggest that *MCEE* inhibition suppresses both proliferation and adipogenic differentiation of porcine intramuscular preadipocytes.

### 3.4. GO and KEGG Enrichment Analyses of DEGs

**Figure 4:**
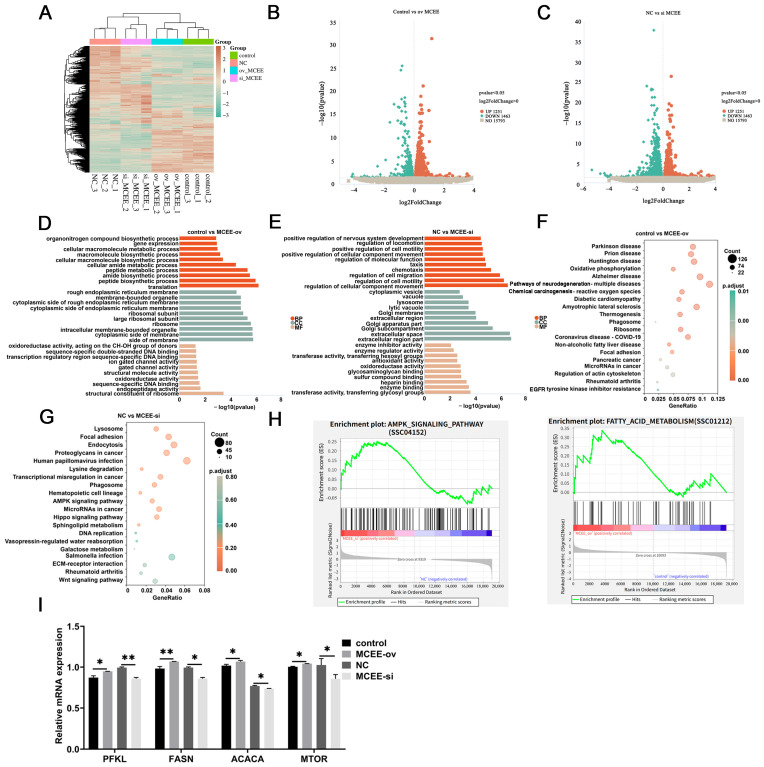
Effects of *MCEE* expression alteration on the transcriptome of intramuscular preadipocytes. (**A**) Heatmap displaying DEGs. (**B**,**C**) Volcano plots illustrating the distribution of DEGs. (**D**,**E**) GO enrichment analysis of DEGs. (**F**,**G**) KEGG pathway enrichment analysis of DEGs. (**H**) The GSEA plots of the fatty acid biosynthesis pathway under *MCEE* gene overexpression and the AMPK signaling pathway under *MCEE* gene inhibition. (**I**) qRT-PCR validation of DEGs. * *p* < 0.05, ** *p* < 0.01.

To investigate the impact of *MCEE* expression on transcriptional regulation, we transfected intramuscular preadipocytes with either pcDNA3.1-*MCEE* overexpression vectors or *MCEE* siRNA, followed by RNA-seq analysis on day 2 of adipogenic differentiation. Heatmap analysis revealed a total of 5034 DEGs screened in both *MCEE* overexpression and knockdown groups (Figure 4A). In the *MCEE* overexpression group, 2320 DEGs were identified, comprising 1278 upregulated and 1042 downregulated genes. Conversely, the *MCEE* knockdown group exhibited 2714 DEGs, with 1251 upregulated and 1463 downregulated genes (Figure 4B,C).

Gene Ontology (GO) and KEGG pathway enrichment analyses were performed on the DEGs identified in *MCEE*-overexpressing and *MCEE*-knockdown groups. GO analysis revealed that DEGs from *MCEE* overexpression or suppression were primarily enriched in biological processes such as oxidoreductase activity and cellular metabolic processes (Figure 4D,E). KEGG analysis indicated that these DEGs were mainly associated with pathways including oxidative phosphorylation, mitochondrial dysfunction-related diseases (such as Parkinson disease, prion disease, and Huntington disease), and the AMPK signaling pathway (Figure 4F,G). These findings suggest that altered *MCEE* expression may be linked to mitochondrial dysfunction. GSEA plots of the fatty acid biosynthesis pathway in *MCEE*-overexpressing cells indicate enhanced fatty acid biosynthesis, whereas GSEA analysis of the AMPK signaling pathway in *MCEE*-knockdown cells demonstrates activation of AMPK signaling upon *MCEE* suppression (Figure 4H). To validate the reliability of the transcriptomic sequencing data, four genes involved in lipid and energy metabolism (*PFKL*, *MTOR*, *FASN*, and *ACACA*) were randomly selected, and their mRNA expression levels were examined. Quantitative results demonstrated that the expression changes in these genes were consistent with the RNA-seq data (Figure 4H), confirming the robustness of the transcriptomic dataset.

### 3.5. MCEE Promotes Mitochondrial Functions

**Figure 5:**
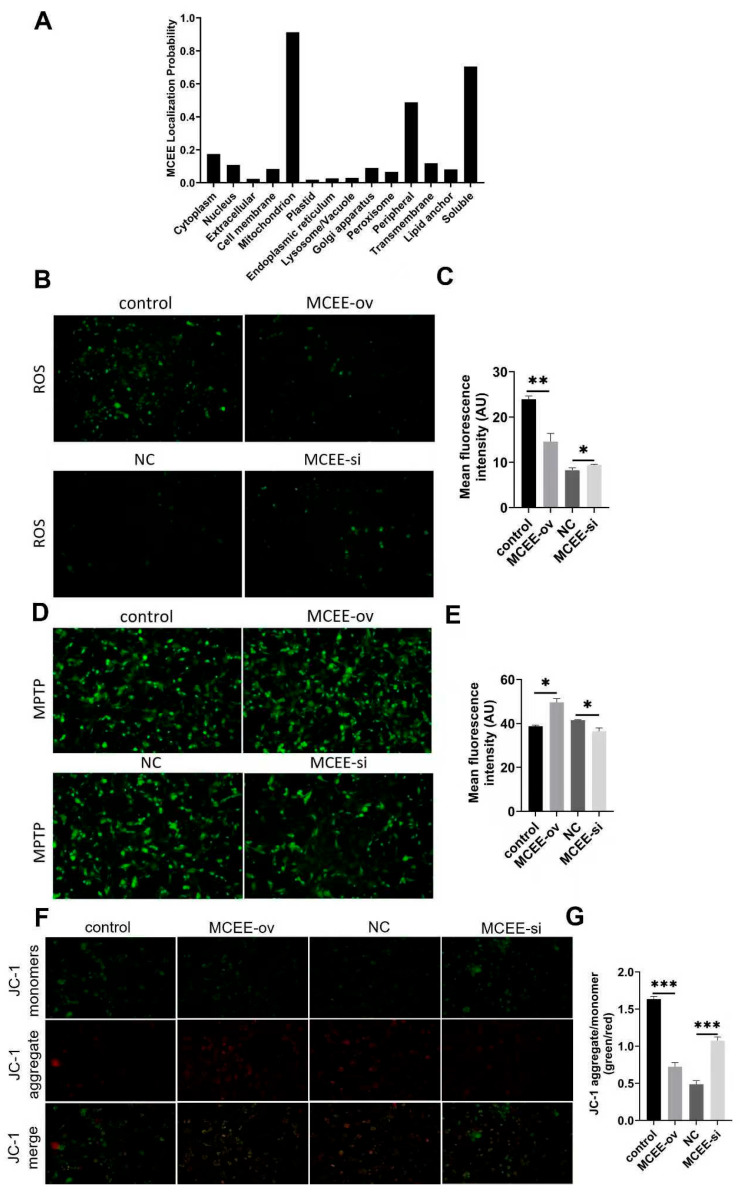
Effects of Altered *MCEE* Expression on Mitochondrial Functions. (**A**) Prediction of *MCEE* subcellular localization. (**B**) Measurement of intracellular ROS levels. (**C**) Quantification of data shown in (**B**). (**D**) mitochondrial permeability transition pore (mPTP) assay, where green fluorescence intensity inversely correlates with mPTP opening. (**E**) Quantification of data shown in (**D**). (**F**) JC-1 fluorescent probe assay for mitochondrial membrane potential. (**G**) Quantification of data shown in (**F**). * *p* < 0.05, ** *p* < 0.01, *** *p* < 0.001.

DEGs resulting from altered *MCEE* expression were primarily enriched in signaling pathways related to oxidative phosphorylation and mitochondrial dysfunction-associated diseases (such as parkinson disease [19], prion disease [20], and huntington disease [21]), as well as the AMPK signaling pathway, suggesting that aberrant *MCEE* expression may be linked to mitochondrial dysfunction. Furthermore, subcellular localization prediction indicated that MCEE is predominantly localized to mitochondria (Figure 5A). We therefore investigated whether altered *MCEE* expression affects mitochondrial function. Intramuscular preadipocytes were transfected with either pcDNA3.1-*MCEE* or *MCEE* siRNA, and mitochondrial parameters were measured two days after inducing adipogenic differentiation. Compared with controls, *MCEE* overexpression significantly reduced intracellular ROS levels, whereas *MCEE* knockdown markedly increased ROS levels (Figure 5B,C), indicating that *MCEE* may help mitigate intracellular ROS accumulation. Additionally, *MCEE* overexpression decreased mitochondrial permeability transition pore (mPTP) opening, while *MCEE* knockdown enhanced its opening (Figure 5D,E). JC-1 staining further revealed that *MCEE* overexpression elevated mitochondrial membrane potential relative to controls, whereas *MCEE* silencing reduced it (Figure 5F,G). These results demonstrate that *MCEE* may promote porcine IMF deposition by regulating mitochondrial function.

## 4. Discussion

In recent years, IMF, as a key determinant influencing pork flavor, tenderness, and juiciness, has garnered extensive attention in both genetic breeding and molecular regulation research [22]. Fat deposition, particularly IMF accumulation, is jointly regulated by multiple factors including signal transduction pathways, transcription factors, metabolic enzymes, non-coding RNAs, and organelle status [23]. The adipogenic differentiation of intramuscular adipocytes is strictly controlled by complex genetic and signaling networks. Our findings demonstrate that *MCEE* participates in regulating the proliferation and adipogenic differentiation of intramuscular preadipocytes, thereby influencing fat deposition.

Gene expression serves as the fundamental basis for functional realization. Overexpression of *MCEE* significantly promoted lipid droplet formation and elevated the transcriptional levels of adipogenic marker genes *FABP4*, *ADIPOQ* and *PLIN1*. These genes represent highly expressed molecular markers during adipocyte maturation, participating in fatty acid transport (*FABP4*), adipocyte metabolism regulation (*ADIPOQ*) and lipid droplet stabilization (*PLIN1*), respectively [24,25]. Their upregulated expression typically reflects the degree of adipocyte differentiation. Concurrently, FABP4 protein expression was significantly elevated following *MCEE* overexpression. Furthermore, Oil Red O staining revealed enhanced lipid droplet accumulation upon *MCEE* overexpression. These results collectively demonstrate the positive regulatory role of *MCEE* in adipocyte differentiation.

Lipid droplets exhibit dynamic alterations in quantity, composition and spatial distribution in proliferating cells, with such dynamics being co-regulated by cell cycle progression to modulate lipid metabolism [26]. Of particular note, both the number and volume of intramuscular adipocytes constitute key determinants of IMF deposition, with cellular quantity and viability being especially critical [27,28]. In this study, viability and apoptosis assays demonstrated that *MCEE* enhances proliferative activity while suppressing apoptosis in intramuscular preadipocytes during early adipogenesis, thereby establishing a quantitative reservoir of precursor cells for adipocyte differentiation. Cell cycle analysis further revealed that *MCEE* overexpression promotes G2/M phase accumulation. This G2/M arrest may reflect a coordinated adaptation of the cell cycle to facilitate adipogenic, as studies have shown that cell cycle arrest at specific phases is often required for initiation of differentiation programs [29].

Fat deposition represents not merely an alteration in cellular morphology and function, but rather a complex process of metabolic reprogramming [30]. To elucidate the molecular mechanisms underlying *MCEE*-mediated fat deposition, we performed transcriptomic profiling. GO and KEGG pathway enrichment analyses revealed that *MCEE*-regulated DEGs were primarily enriched in biological processes such as oxidoreductase activity and cellular metabolic processes, and were predominantly associated with pathways including oxidative phosphorylation, mitochondrial dysfunction-related disorders (such as parkinson disease, prion disease, and huntington disease), as well as the AMPK signaling pathway. Furthermore, subcellular localization prediction indicated that MCEE is primarily localized to mitochondria. These findings suggest that altered *MCEE* expression may be linked to mitochondrial dysfunction. The enrichment of mitochondrial dysfunction-related pathways highlights the potential role of *MCEE* in maintaining mitochondrial homeostasis, which is crucial for adipocyte differentiation and function.

AMPK promotes mitochondrial biogenesis in response to cellular energy deficiency. When the ATP/AMP ratio decreases, AMPK is activated to facilitate mitochondrial production, a process involving the regulation of *PGC1α* and *CaMKIV* [31]. After inhibiting *MCEE* expression, KEGG analysis revealed significant enrichment of the AMPK signaling pathway, suggesting a potential low-energy state in cells. The AMPK and MTORC1 pathways dynamically regulate cellular energy status and growth requirements through mutual antagonism [32,33,34]. The MTORC1 pathway can significantly upregulate the expression of propionate metabolism-related genes (including *MCEE*, *PCCA*, and *MMUT*) by activating *PGC1α*, thereby enhancing the overall efficiency of the propionate metabolic pathway and indirectly promoting pyruvate production [15]. These observations suggest a feedback mechanism wherein *MCEE* downregulation leads to AMPK activation, possibly due to impaired propionate metabolism and reduced energy output. This is consistent with reports that disruptions in mitochondrial metabolite processing can activate energy-sensing pathways like AMPK [35]. These pathways are widely recognized as central to adipogenesis, lipid droplet formation, energy sensing, and metabolic regulation, implying that *MCEE* may coordinately regulate fat deposition through multiple signaling and metabolic networks.

Altered *MCEE* expression levels significantly modulated multiple metabolism-related genes, including fatty acid synthesis genes (*ACACA*, *FASN*, and *ACLY*), glycolysis and energy metabolism gene (*PFKL*), and MTOR signaling pathway gene (*MTOR*), all of which play pivotal roles in adipocyte differentiation and functional maintenance. *ACACA* and *FASN* encode acetyl-CoA carboxylase and fatty acid synthase, respectively, key rate-limiting enzymes in de novo fatty acid synthesis. Elevated expression of these genes typically indicates enhanced fatty acid synthesis capacity [36]. With NADPH participation, FAS catalyzes the de novo synthesis of long-chain saturated fatty acids using acetyl-CoA as a substrate [37]. Our data demonstrated upregulation of *FASN* in *MCEE*-overexpressing groups and downregulation in *MCEE*-suppressed groups, further validating *MCEE*’s potential role in promoting fatty acid synthesis. These findings suggest that *MCEE* may facilitate IMF deposition by orchestrating the expression of these critical metabolic enzymes.

As the energy hub of adipocyte metabolism, the functional status of mitochondria directly influences fatty acid synthesis, transport, and degradation [30,38]. By predicting the subcellular localization of *MCEE*, we found that it is primarily located in mitochondria. Therefore, we also investigated the role of *MCEE* in maintaining mitochondrial function in adipocytes. In cells overexpressing *MCEE*, the levels of ROS were significantly reduced, accompanied by a decline in mitochondrial membrane potential and decreased opening of the mitochondrial permeability transition pore (mPTP), suggesting that *MCEE* sustains mitochondrial function. The reduction in ROS, coupled with improved mitochondrial membrane integrity, implies that *MCEE* may help mitigate oxidative damage, which is known to impair adipogenic differentiation [39]. The observed changes in mPTP opening are particularly relevant, as prolonged mPTP opening can lead to mitochondrial swelling and apoptosis, thereby negatively affecting adipocyte survival and lipid accumulation [40]. This state promotes fatty acid synthesis and lipid droplet accumulation, creating a metabolic environment essential for adipocyte differentiation. Conversely, inhibition of *MCEE* induces mitochondrial dysfunction in cells, which may impair energy metabolism and exacerbate oxidative stress in adipocytes, thereby suppressing lipogenesis.

In summary, this study systematically reveals, for the first time, the important mechanistic role of *MCEE* in porcine IMF deposition. *MCEE* promotes lipid droplet accumulation and adipogenic phenotype formation by regulating the proliferation and differentiation of intramuscular preadipocytes, maintaining mitochondrial function stability, reducing oxidative stress levels. These findings not only expand our understanding of the regulatory network of IMF deposition but also provide potential target genes and theoretical foundations for molecular breeding of pork quality traits.

## 5. Conclusions

Our study demonstrates that *MCEE* plays a pivotal role in regulating porcine IMF deposition by modulating mitochondrial function in preadipocytes. Through gain- and loss-of-function experiments, we established that *MCEE* enhances adipogenic differentiation and cell proliferation, with transcriptomic analysis linking its activity to oxidative phosphorylation and mitochondrial functions. Furthermore, *MCEE* influences critical mitochondrial parameters, including ROS levels, membrane potential, and permeability transition pore opening, underscoring its mechanistic involvement in IMF accumulation. These findings not only advance our understanding of the molecular basis of IMF formation but also highlight *MCEE* as a promising target for genetic or nutritional strategies aimed at improving meat quality in livestock, particularly in pig production.

## Figures and Tables

**Table 1:** Primer sequences for RT-qRCR.

Gene	Primer Sequences
*MCEE*	Forward 5′-TCCTGCTGAGAACGATGGAAG-3′
	Reverse 5′-GATTGAGTCGCCCCAGGTTC-3′
*ADIPOQ*	Forward 5′-ATGTCTACCGTTCAGCATTCA-3′
	Reverse 5′-GAGTACAGCCTTGTCCTTCTTG-3′
*FABP4*	Forward 5′-AAATACTGAGATTGCCTTCA-3′
	Reverse 5′-ACATTCCACCACCAACTTAT-3′
*PLIN1*	Forward 5′-CCCTGGTGGCGTCTGTATG-3′
	Reverse 5′-GGAGGCGGGTGGAGATTGT-3′
*FASN*	Forward 5′-CGTTGGGTCGACTCACTGAA-3′
	Reverse 5′-GAGACAGTTCACCATGCCCA-3′
*ACACA*	Forward 5′-GGGAACATCCCCACGCTAAA-3′
	Reverse 5′-GAAAGAGACCATTCCGCCCA-3′
*PKFL*	Forward 5′-ATGGCTACCGTGGACCTGGAGAA-3′
	Reverse 5′-TTGATGTTCTCGCCTCCTTCC-3′
*MTOR*	Forward 5′-GACGGATTCCTACTCTGCCG-3′
	Reverse 5′-TTTAGGGCCTCCGGTTTCAC-3′
*β-actin*	Forward 5′-CTGGCACCACACCTTCTACAA-3′
	Reverse 5′-GTGTTGAAGGTCTCGAACATGAT-3′

## Data Availability

The data that support the findings of this study are available from the corresponding author upon reasonable request.

## Supplementary Materials

The following supporting information can be downloaded at: https://www.mdpi.com/article/10.3390/ani15192797/s1, Western blot original images.
