# Supplementary figures and images for: MCEE Promotes Intramuscular Fat Deposition in Pigs Through Regulating Mitochondrial Function
Source: Animals (Basel). 2025 Sep 25;15(19):2797. doi: 10.3390/ani15192797 (PMC12523552; doi:10.3390/ani15192797)

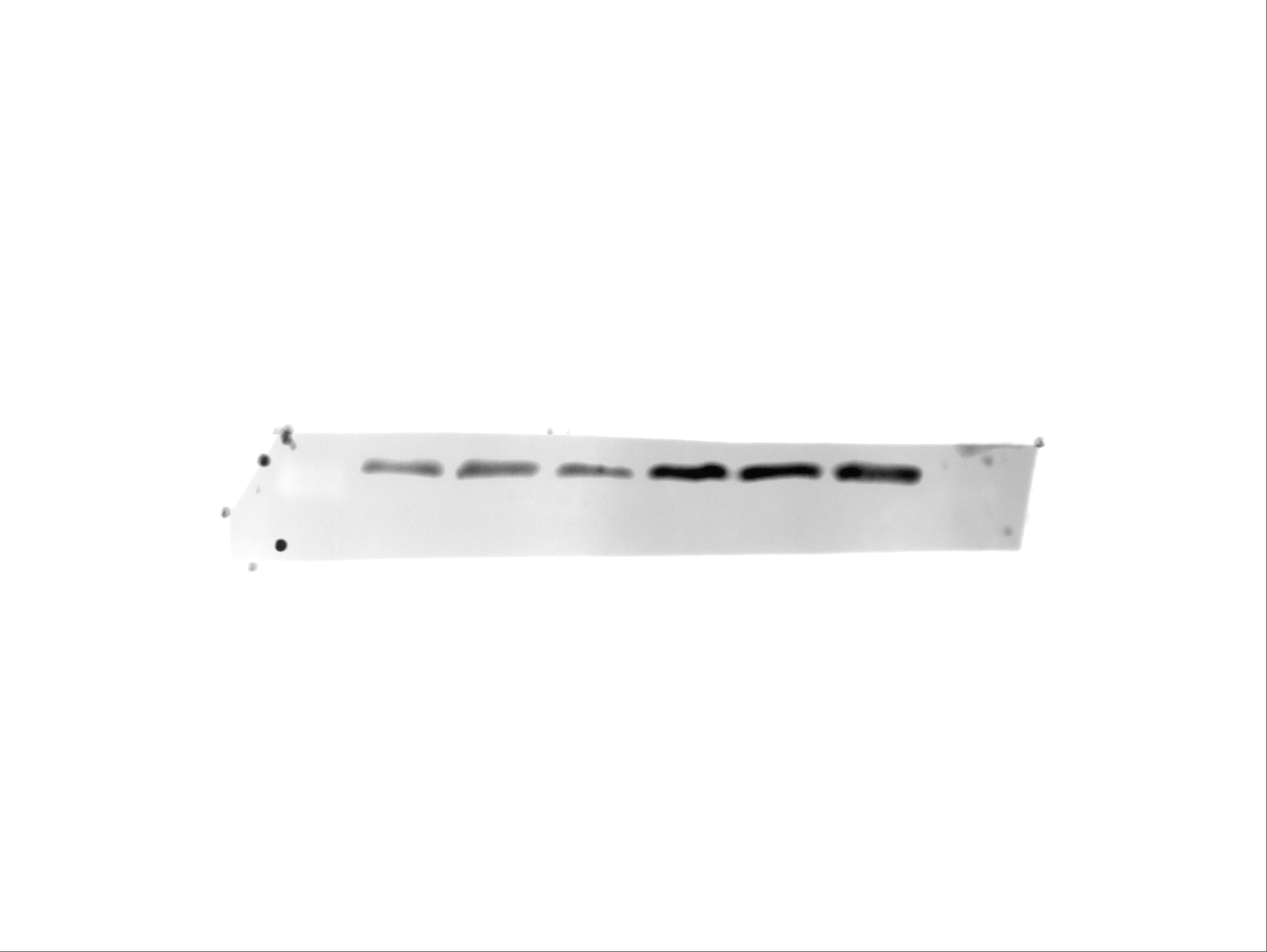

Supplement: Supplementary file 1 [file animals-15-02797-s001.zip › animals-3841905-SI/Western blot original image/The original WB image of Figure 2B/FABP4.tif]

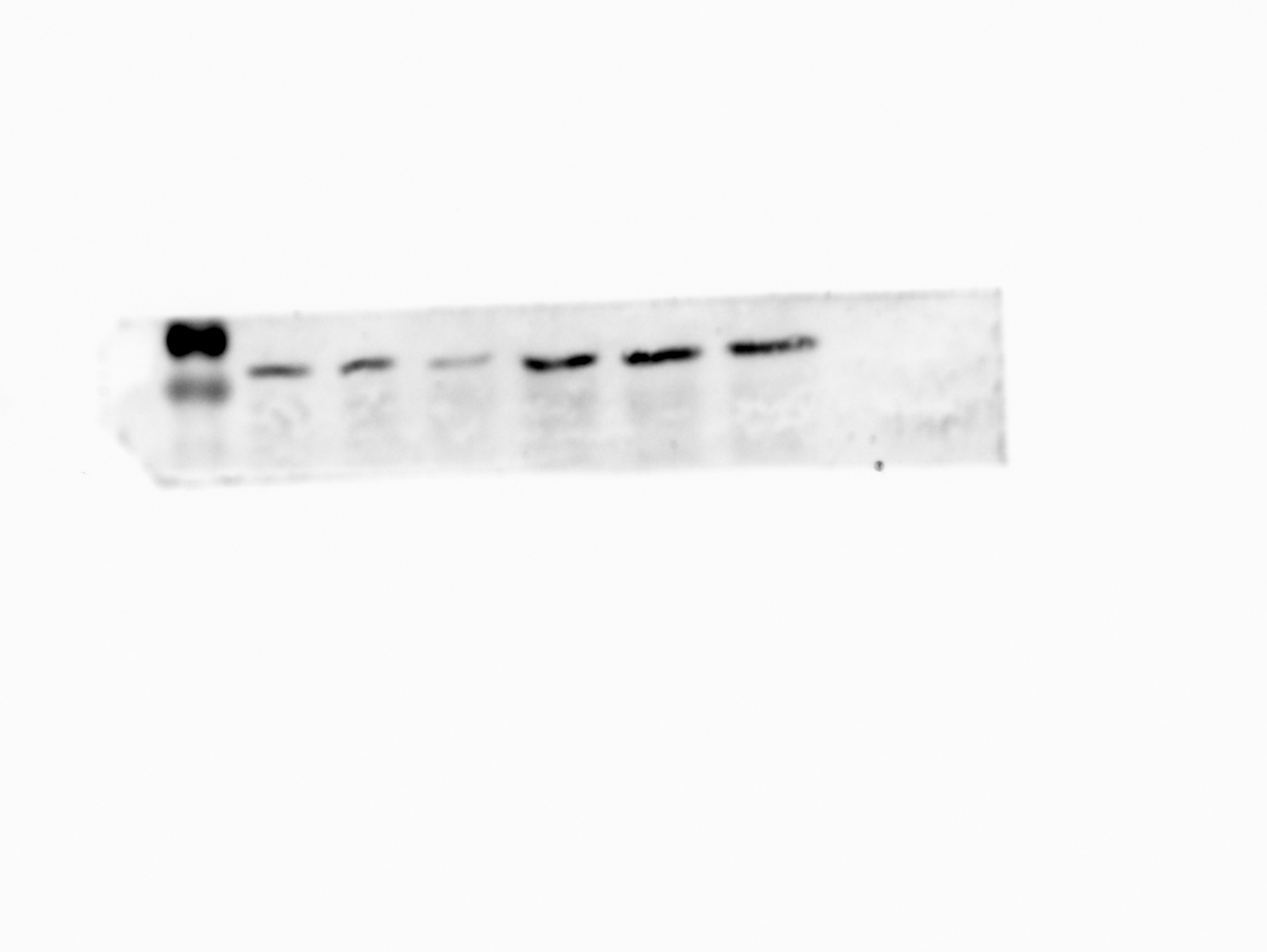

Supplement: Supplementary file 1 [file animals-15-02797-s001.zip › animals-3841905-SI/Western blot original image/The original WB image of Figure 2B/MCEE.tif]

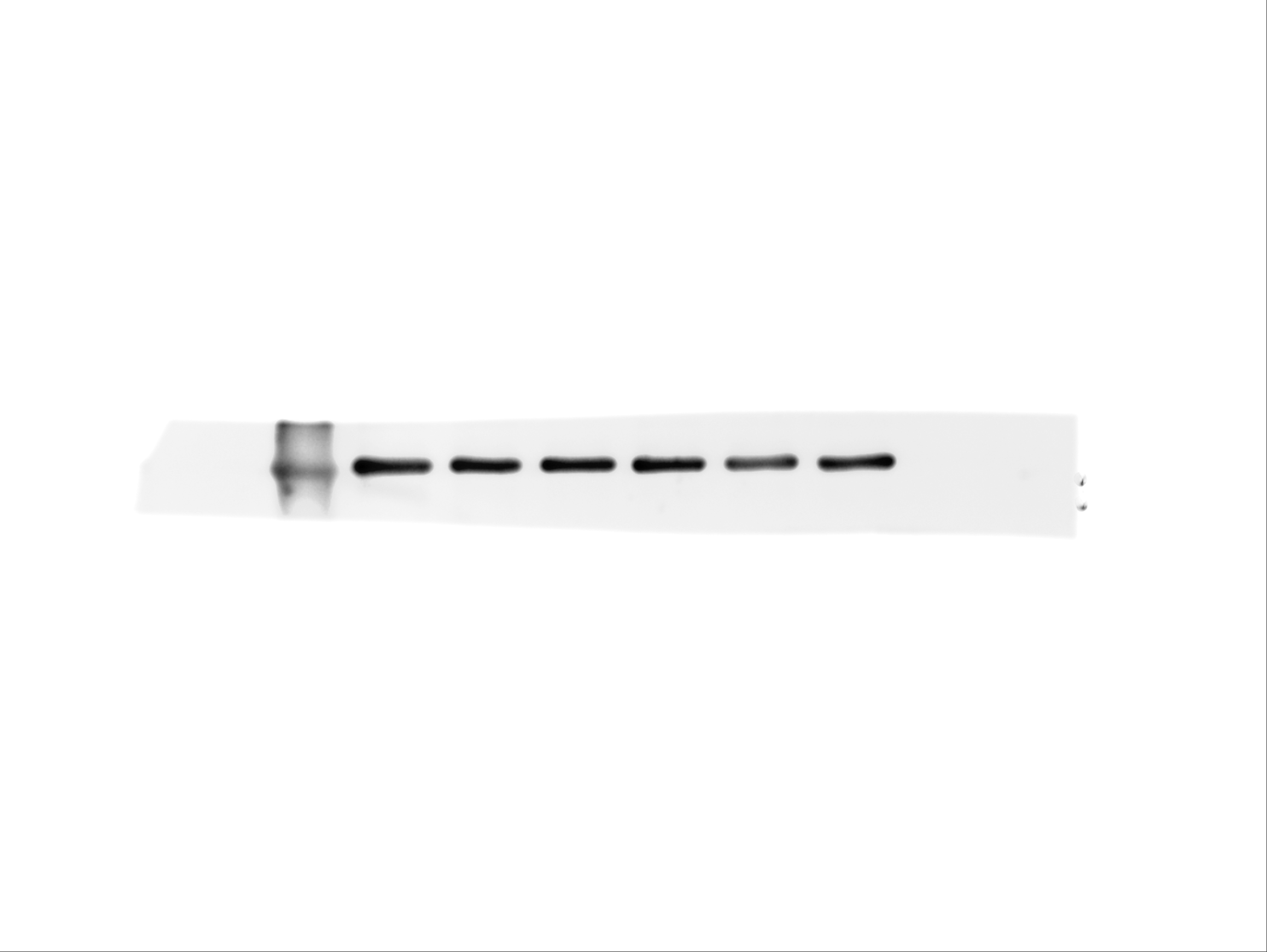

Supplement: Supplementary file 1 [file animals-15-02797-s001.zip › animals-3841905-SI/Western blot original image/The original WB image of Figure 2B/β-actin.tif]

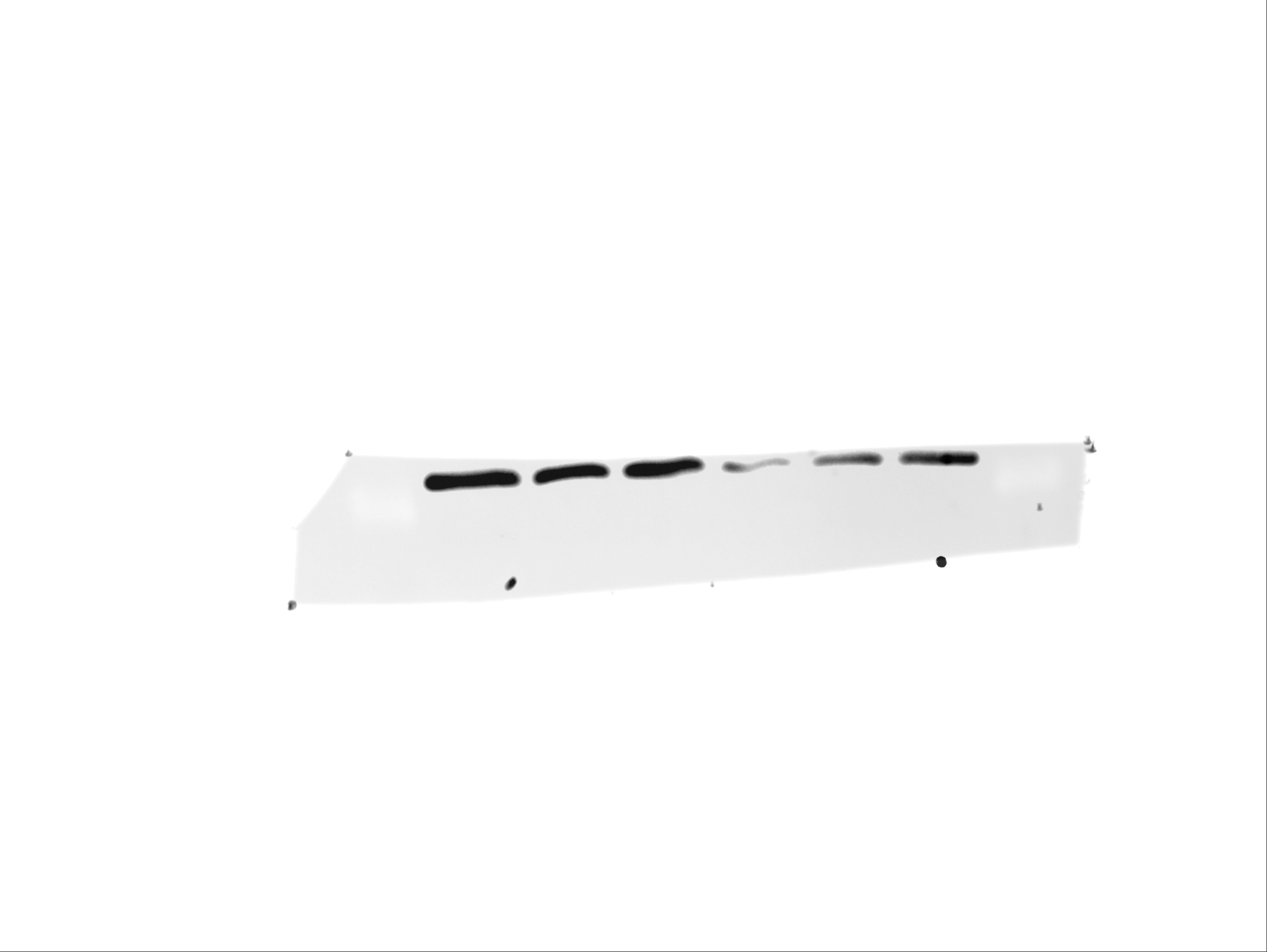

Supplement: Supplementary file 1 [file animals-15-02797-s001.zip › animals-3841905-SI/Western blot original image/The original WB image of Figure 3B/FABP4.tif]

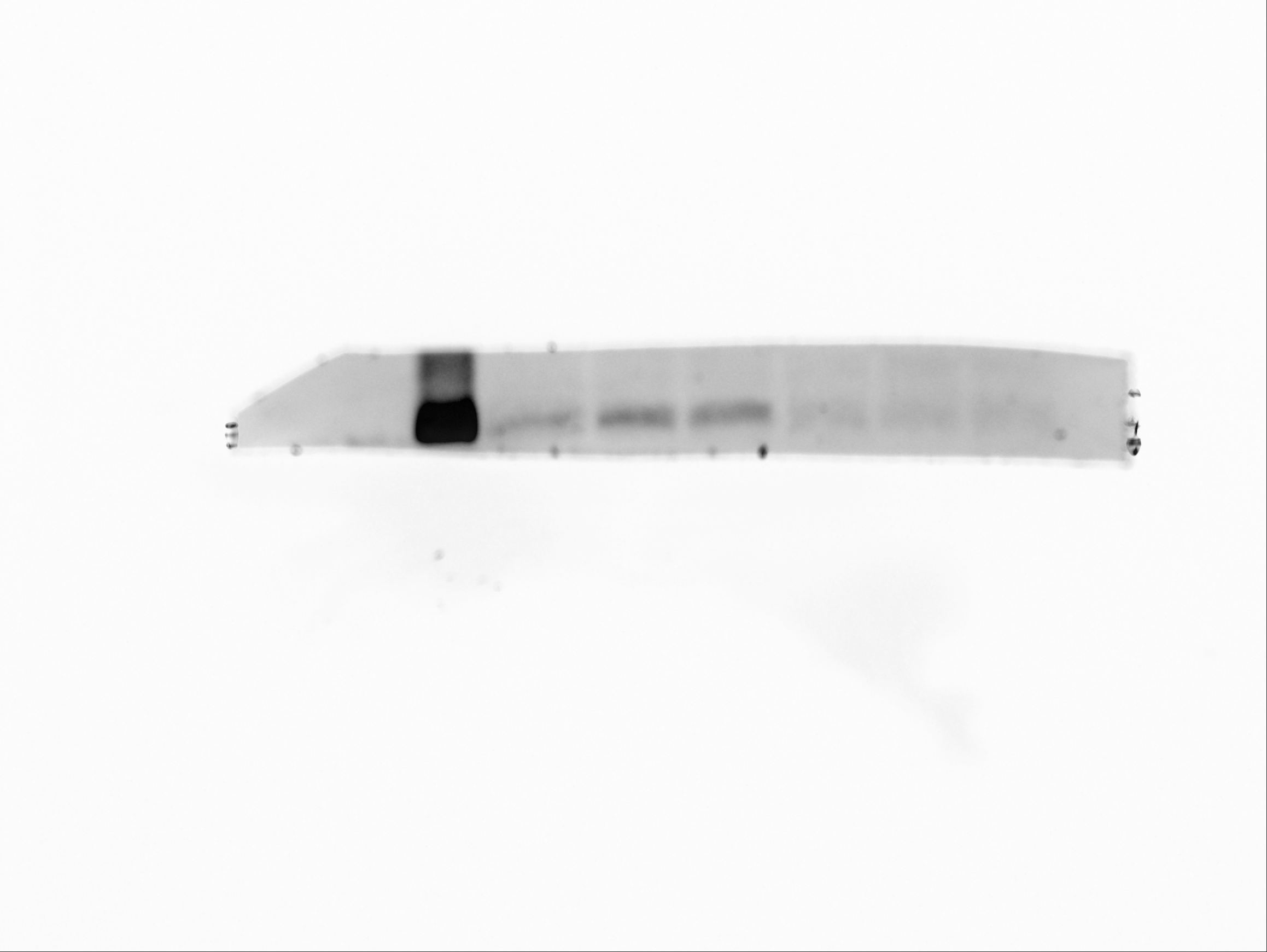

Supplement: Supplementary file 1 [file animals-15-02797-s001.zip › animals-3841905-SI/Western blot original image/The original WB image of Figure 3B/MCEE.tif]

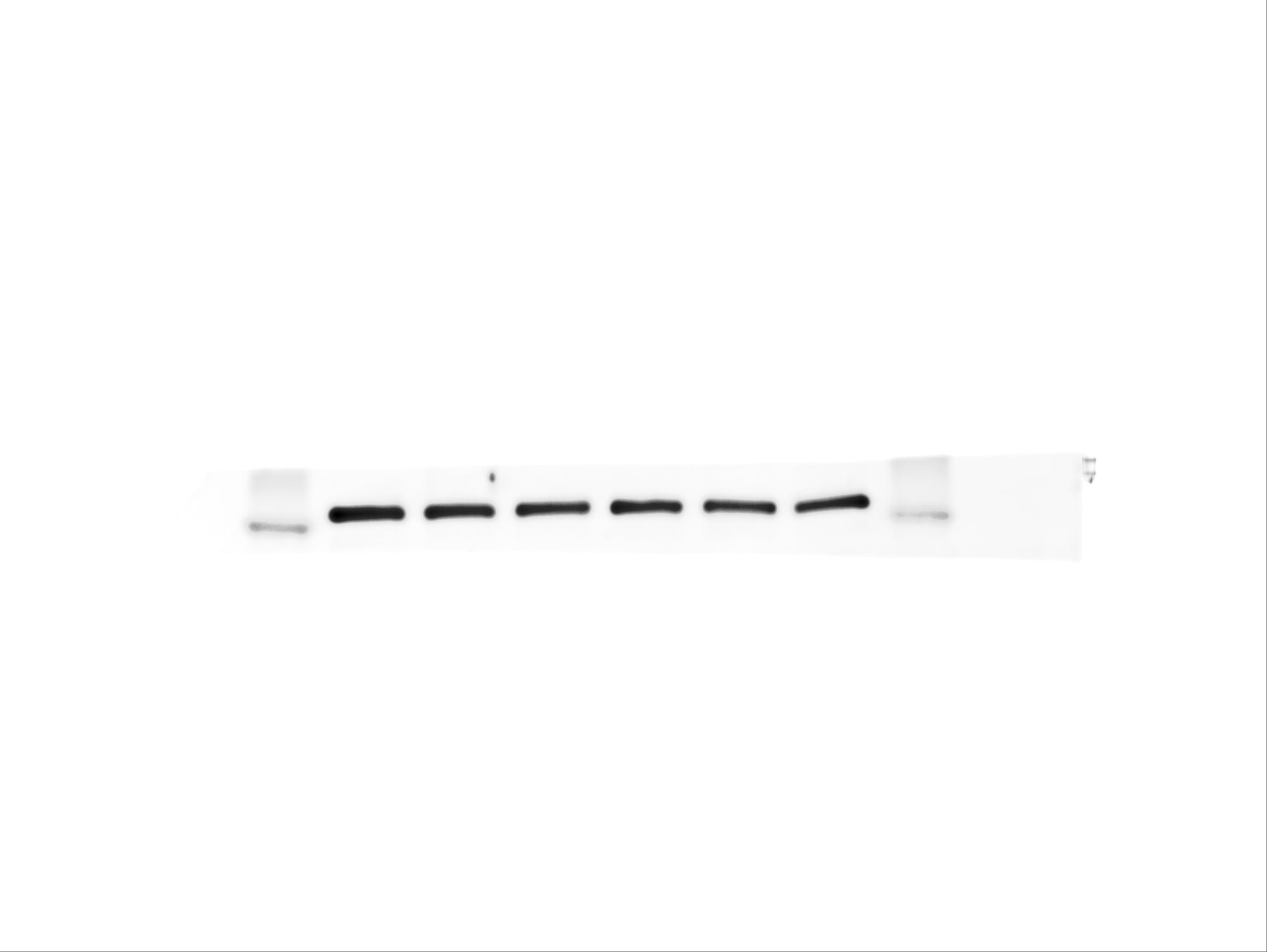

Supplement: Supplementary file 1 [file animals-15-02797-s001.zip › animals-3841905-SI/Western blot original image/The original WB image of Figure 3B/β-actin.tif]
